# Microbiota-based analysis reveals specific bacterial traits and a novel strategy for the diagnosis of infectious infertility

**DOI:** 10.1371/journal.pone.0191047

**Published:** 2018-01-09

**Authors:** Simon Graspeuntner, Michael K. Bohlmann, Kathrin Gillmann, Runa Speer, Sven Kuenzel, Heike Mark, Friederike Hoellen, Reinhard Lettau, Georg Griesinger, Inke R. König, John F. Baines, Jan Rupp

**Affiliations:** 1 Department of Infectious Diseases and Microbiology, University of Luebeck, Luebeck, Schleswig-Holstein, Germany; 2 Department of Obstetrics and Gynecology, University Hospital of Schleswig-Holstein, Luebeck, Schleswig-Holstein, Germany; 3 Department of Obstetrics and Gynecology, University Hospital Mannheim, Mannheim, Baden-Wuertemberg, Germany; 4 Berlin Department of Public Health, Center for Sexual Health, Berlin, Berlin, Germany; 5 Max Planck Institute for Evolutionary Biology, Ploen, Schleswig-Holstein, Germany; 6 Outpatient Medical Care for Gynecology, Luebeck, Schleswig-Holstein, Germany; 7 Department of Reproductive Medicine and Gynecological Endocrinology, University Hospital of Schleswig-Holstein, Luebeck, Schleswig-Holstein, Germany; 8 Institute of Medical Biometry and Statistics, University of Luebeck, Luebeck, Schleswig-Holstein, Germany; 9 Institute for Experimental Medicine, Christian-Albrechts-University of Kiel, Kiel, Schleswig-Holstein, Germany; Children's Hospital Oakland Research Institute, UNITED STATES

## Abstract

Tubal factor infertility (TFI) accounts for more than 30% of the cases of female infertility and mostly resides from an inflammatory process triggered by an infection. Clinical appearances largely differ, and very often infections are not recognized or remain completely asymptomatic over time. Here, we characterized the microbial pattern in females diagnosed with infectious infertility (ININF) in comparison to females with non-infectious infertility (nININF), female sex workers (FSW) and healthy controls (fertile). Females diagnosed with infectious infertility differed significantly in the seroprevalence of IgG antibodies against the *C*. *trachomatis* proteins MOMP, OMP2, CPAF and HSP60 when compared to fertile females. Microbiota analysis using 16S amplicon sequencing of cervical swabs revealed significant differences between ININF and fertile controls in the relative read count of *Gardnerella* (10.08% *vs*. 5.43%). Alpha diversity varies among groups, which are characterized by community state types including *Lactobacillus*-dominated communities in fertile females, an increase in diversity in all the other groups and *Gardnerella*-dominated communities occurring more often in ININF. While all single parameters did not allow predicting infections as the cause of infertility, including *C*. *trachomatis* IgG/IgA status together with 16S rRNA gene analysis of the ten most frequent taxa a total of 93.8% of the females were correctly classified. Further studies are needed to unravel the impact of the cervical microbiota in the pathogenesis of infectious infertility and its potential for identifying females at risk earlier in life.

## Introduction

Tubal factor infertility is a major cause of involuntary childlessness in females that mostly derives from an inflammatory process in the fallopian tubes caused by a sexual-transmitted infection (STI). Epidemiological data suggest *Chlamydia trachomatis* and *Neisseria gonorrhoeae* as the most prevalent STIs worldwide, however, direct correlation between the STI and infertility is hampered by several limitations: 1) most acute STI are asymptomatic and are not recognized, 2) diagnostic approaches often miss chronic infections, 3) there is often a long time span between an acute STI-related inflammatory disease (e.g. pelvic inflammatory disease, salpingitis) and the recognition of female infertility.

The current diagnostic workflow in detecting infection as the underlying reason for female infertility comprises anamnestic and serological analysis on previous STIs, combined with diagnostic assessments of anomalies of the reproductive tract of both partners [1]. Limitations in detecting previous STIs that often remained asymptomatic during acute infection, but also discrepancies between the retrieval of pathogens from the lower and upper genital tract by cultivation and nucleic acid detection, makes it difficult in the clinical routine to identify all cases of infectious infertility. The emergence of bacterial vaginosis (BV) is considered as one of the key factors in the development of female diseases of the upper genital tract and has been associated with pre-term birth, salpingitis and pelvic inflammatory disease [2]. However, the role of BV in this context is not well understood, arguing for clinical, inflammatory and microbial patterns to be considered in the link between BV and long term sequels of STIs. Recently, a shift in the microbial composition of the female genital tract, which is accompanied by a decrease of *Lactobacillus*-dominated communities, has been determined as one of the hallmarks of BV in different clinical entities [3, 4]. Furthermore, high diversity communities including high relative abundance of *Gardnerella* and *Ureaplasma* in the vaginal tract have been shown to be associated with preterm birth [5]. In general, next-generation-sequencing techniques offer a new approach in studying bacterial-bacterial association within the urogenital microbiome [6] in comparison to classical, cultivation-dependent methods. Clinical studies in females suffering from infectious infertility that integrate data from vaginal microbiota sequencing are not yet performed. By overcoming the limitations of classical studies, recent research highlights the importance of the cervico-vaginal microbiota in reproductive health, e.g. through the inhibition of pathogens such as *E*. *coli* [7], HIV [8] *C*. *trachomatis* [9]. Besides others, *L*. *crispatus* dominated vaginal bacterial communities have been described to be protective in the transmission of *C*. *trachomatis* from infected men to women [10]. As outlined in a recent review of Ziklo et al. a diverse microbial community is suggested to provide a particular microenvironment favoring the growth of *C*. *trachomatis* [11], thereby playing an important role in development of infectious infertility. However, clinical studies in females suffering from infectious infertility that integrate data from vaginal microbiota sequencing are not yet performed. Here, we characterized the microbial pattern found in females with infectious infertility in comparison to females with non-infectious infertility, fertile controls and female sex workers. The aims of this study were to obtain a detailed insight into the bacterial burden and communities of females at the time of diagnosis of infectious infertility, and propose a diagnostic algorithm for identification of females at risk during earlier screening.

## Materials and methods

### Study design and sample collection

From 2012 to 2014 female patients from the Infertility Clinic at the University Hospital Schleswig-Holstein/Campus Luebeck, fertile controls from an Outpatient Clinic in Luebeck and female sex workers (FSW) from the Center of Sexual Health in Berlin were prospectively included in the study. The work on human samples within this study was approved by the ethics commitee of the University of Lübeck at the 05.02.2012 with the reference number 11–185. All participants were informed and signed a written consent. Females from the Infertility Clinic were classified by the treating physicians into the two groups, infectious infertility (ININF) and non-infectious infertility (nININF), after finalization of the conventional diagnostic assessment and before the samples were further processed. Three swabs from the cervix uteri were obtained from every woman by a trained gynecologist, avoiding contamination from the lower genital tract. Elution-swab samples (Copan) were used for subsequent storage in Amies transport medium (Copan) or UTM- medium (Copan) and transferred to the local microbiology laboratory. In addition, 10 ml of serum was collected from every women included in the study for testing anti-chlamydial antibodies. Only women with a complete set of swabs and serum were finally included in the analysis.

### Processing of the cervical swabs and the serum

The first cervical swab was used for conventional bacterial culture of commensal and pathogenic bacteria of the urogenital tract. According to the standard operating procedures, the samples were plated on blood and chocolate agar plates and incubated for up to 48 hours. The second swab was stored at -80°C until direct PCR-testing against *C*. *trachomatis*, *N*. *gonorrhoeae*, *M*. *genitalium*, *M*. *hominis* and *U*. *urealyticum* (TIB Molbiol) was performed. The third swab was also stored at -80°C until further processing for the 16S rRNA gene sequencing. In addition, serum samples were tested with an ELISA (Mikrogen) and Immunblot Assay (Mikrogen) to specifically determine *C*. *trachomatis* IgG and IgA- antibodies against the chlamydial proteins MOMP, OMP2, TARP, CPAF and HSP60.

### DNA-extraction

Swabs in UTM™ media (Copan Diagnostics) were vortexed for 1 minute on maximum speed. Bacterial DNA was isolated from 1 ml media using PowerSoil® DNA Isolation Kit (MoBio) according to the manufacturer´s protocol. The method was modified by using double amounts of solutions C2, C3 and C4 and a 2h incubation with OB Protease (Peqlab) at 50°C, followed by a homogenization step prior to the first centrifugation step. A negative extraction control was run for each extraction.

### Amplicon generation, preparation and sequencing

Partial sequences of the bacterial 16S rRNA gene were amplified with primers spanning the V3/V4 hypervariable regions adopted from Kozich et. al. [12]. The primers contained unique indices and linker sequences for paired end sequencing on an lllumina® MiSeq sequencer. PCR was performed as follows: 98°C for 5 min followed by 30 cycles with 98°C for 9 sec, 55°C for 60 sec and 72°C for 90 sec followed by a final step at 72°C for 10 min. After PCR samples were stored at -20°C until further usage. Amplicons were quantified on an agarose gel with a DNA ladder as reference, were the concentration of each amplicon was determined by comparison to a ladder band of the same size and intensity as the respective amplicon. Equimolar amounts of the correct sized fragments were pooled for sequencing. Afterwards the pool was run again on an agarose gel and eluated with MinEluteGel Extraction Kit (Qiagen). The pool was stored at -20°C until sequencing. Sequencing was performed on a MiSeq sequencer (Illumina^®^) using the MiSeq Reagent Kit v3 (600 cycles) as described by Kozich et. al. [12].

### Data processing

Fastq files were processed using mothur version 1.35.0 [13]. Contigs were produced of forward and reverse sequences and any sequence was removed if it had ambiguous bases, a homopolymer length > 9 or a size longer than the amplified fragment. We aligned the remaining sequences using a customized SILVA reference data base [14] and removed unaligned sequences. Chimeras were detected using the UCHIME algorithm [15] as implemented in mothur [13] and removed from the data set. We classified the sequences using the mothur-formatted RDP [16] training set version 9, with a cutoff of 80 and removed non-bacterial sequences. Further analysis was done on a random subset of 2500 reads/sample, either using operational taxonomic units (OTUs) clustered with a similarity threshold of 97% or based on taxonomic assignment. To keep a consistent classification on the genus and species level, we used Stirrups for taxonomic assignment with a reference taxonomy specifically produced for vaginal microbiota [17]. We used PICRUSt (Phylogenetic Investigation of Communities of Unobserved States) as online tool (http://huttenhower.sph.harvard.edu/galaxy/) to predict the metagenome of microbial communities [18].

### Statistical analysis

Statistics and graphical visualizations were produced using R version 3.2.2 [19]. We tested the prevalence of diagnostic parameters globally over all 4 groups using Pearson´s Chi-square tests with Holm correction for the number of calculations within each set of diagnostic tests (PCR-testing, cultivation of pathogens, IgG-serology and IgA serology). Subsequently, we used Fisher´s exact tests to determine which groups differ significantly from each other in each parameter. The relative read count of bacterial taxa from the sequencing was calculated per group and differences between the groups were tested using Kruskal-Wallis rank sum tests with Benjamini-Hochberg correction for the number of taxa tested. Subsequent pairwise comparisons were performed for significant taxa using Wilcoxon rank sum test with continuity correction. Assessing bacterial alpha diversity, we calculated Simpson´s diversity index and Shannon´s diversity index for each sample on the basis of OTUs using R package vegan [20]. Differences in alpha diversity between groups were tested based on Simpson´s diversity index using the Kruskal-Wallis rank sum test followed by pairwise Wilcoxon rank sum test with Benjamini-Hochberg correction. We tested the influence of sexual intercourse on alpha diversity based on Shannon´s diversity index. Therefore we used fractional polynomials to assess the best regression model [21]. Based on the best proposed model we performed linear regression and computed Pearson´s product-moment correlation. A heatmap of the 25 main taxa from the sequencing was produced using R package BoutrosLab.plotting.general [22]. Furthermore, bacterial communities were clustered into community state types (CST). CSTs were determined using euclidean distances with complete linkage clustering. The observed CSTs were named on the basis of the dominant member of the respective CST. Differences in the proportion of the community types were assessed using the Chi-square test.

### Probability modelling

We established a prediction model for suffering from infectious infertility based on a subset of the clinical and sequencing data excluding FSW. Prediction was performed using binary logistic regression, and the following parameters were included as predictors in the modelling: detection of bacterial pathogens by culture/PCR, IgA and IgG- immunoblots against epitopes of *C*. *trachomatis*, and the most abundant 10 taxa from the sequencing analysis. Samples were assigned as suffering from infectious infertility if they reached a probability threshold of 0.2 while other samples were assigned as not suffering from infectious infertility. We assessed the prediction outcome and the total accuracy of the model. Goodness-of-fit of the model was assessed using McFadden`s pseudo R^2^. ROC curves were generated assessing true positive rate versus false positive rate and the area under curve and its 95% confidence interval (CI) was computed using r-packages ROCR [23] and pROC [24].

### Accession numbers

The datasets analyzed during the current study are available at the European Nucleotide Archive (ENA) with the accession number PRJEB17077.

## Results

From 2012 to 2014, a total of 210 females were recruited for this study. Within the couples from the infertility group, 26 females were classified as having non-infectious infertility (nININF) on the basis of the currently recommended diagnostic assessment [1]. The main reasons for nININF included diagnosis of Uterus myomatosus, polycystic ovarian syndrome (PCOS), endometriosis or proven infertility of the male partner. 21 females were classified as having infectious infertility (ININF), including females with a history of pelvic inflammatory disease (PID), and females with tubal occlusion due to acute or chronic PID. 89 fertile females were included on the basis of having children and no evidence of current STI. In addition, 54 female sex workers (FSW) were randomly included in this study for adjusting the results with respect to frequencies of sexual intercourse and underlying STIs.

### History of previous STIs and detection of pathogens from cervical swabs

**Fig 1:**
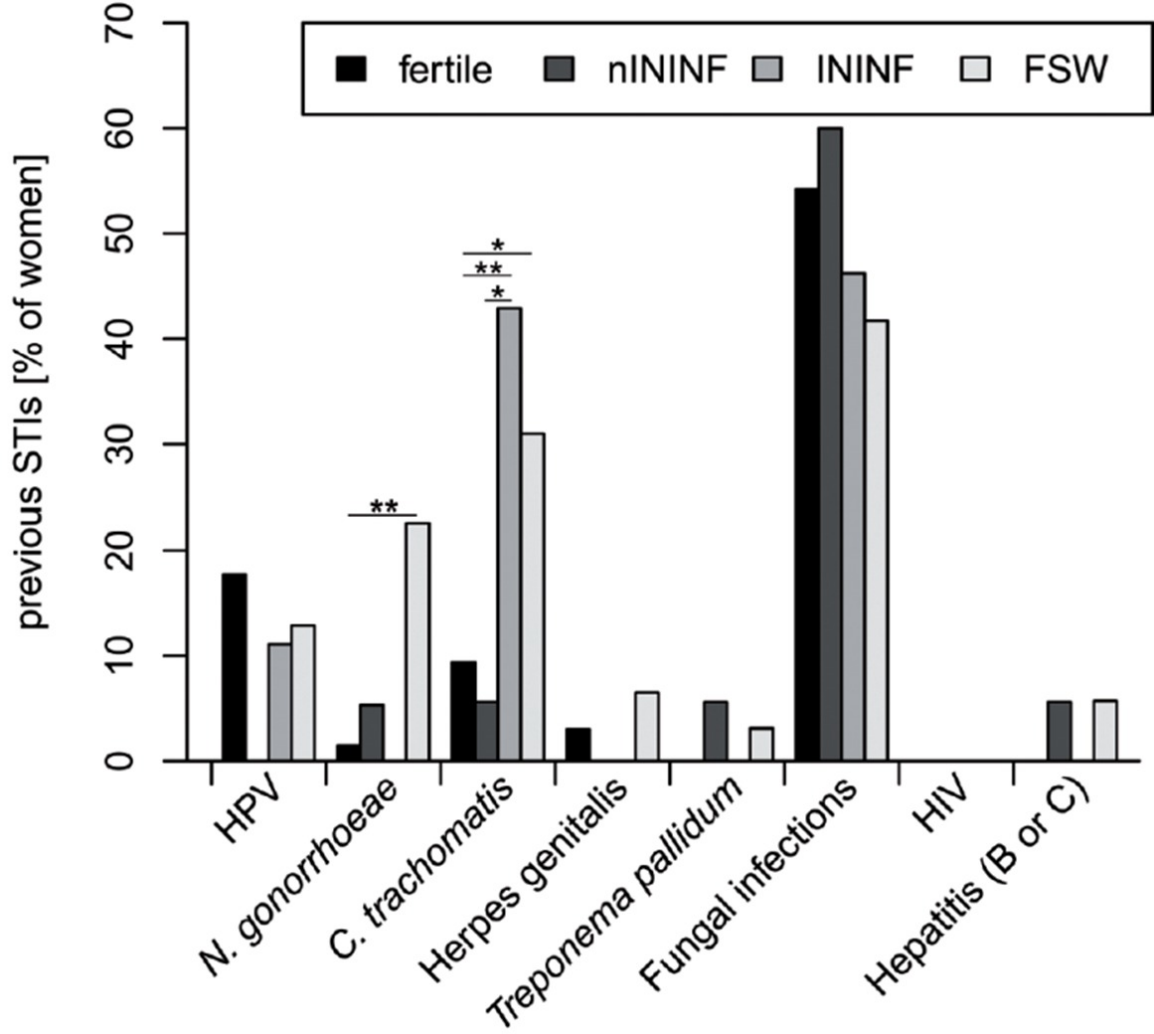
Self-reported questionnaire on previous infections. Including human Papillomavirus (HPV), *N*. *gonorrhoeae*, Herpes simplex virus (HSV), *Treponema pallidum*, Hepatitis B/C and HIV (Fisher´s exact test: *p<0.05, **p<0.01).

In the group of female sex workers (FSW) significantly more infections with *C*. *trachomatis* and *N*. *gonorrhoeae* are reported when compared to fertile controls (Fig 1). Females with infectious infertility (ININF) significantly differ in the frequency of previous *C*. *trachomatis* infections in comparison to fertile controls (p<0.01) and females with nININF (p<0.05). No differences between the groups are observed for previous infections with HPV, HSV, *Treponema pallidum*, HIV or Hepatitis B and C.

**Fig 2:**
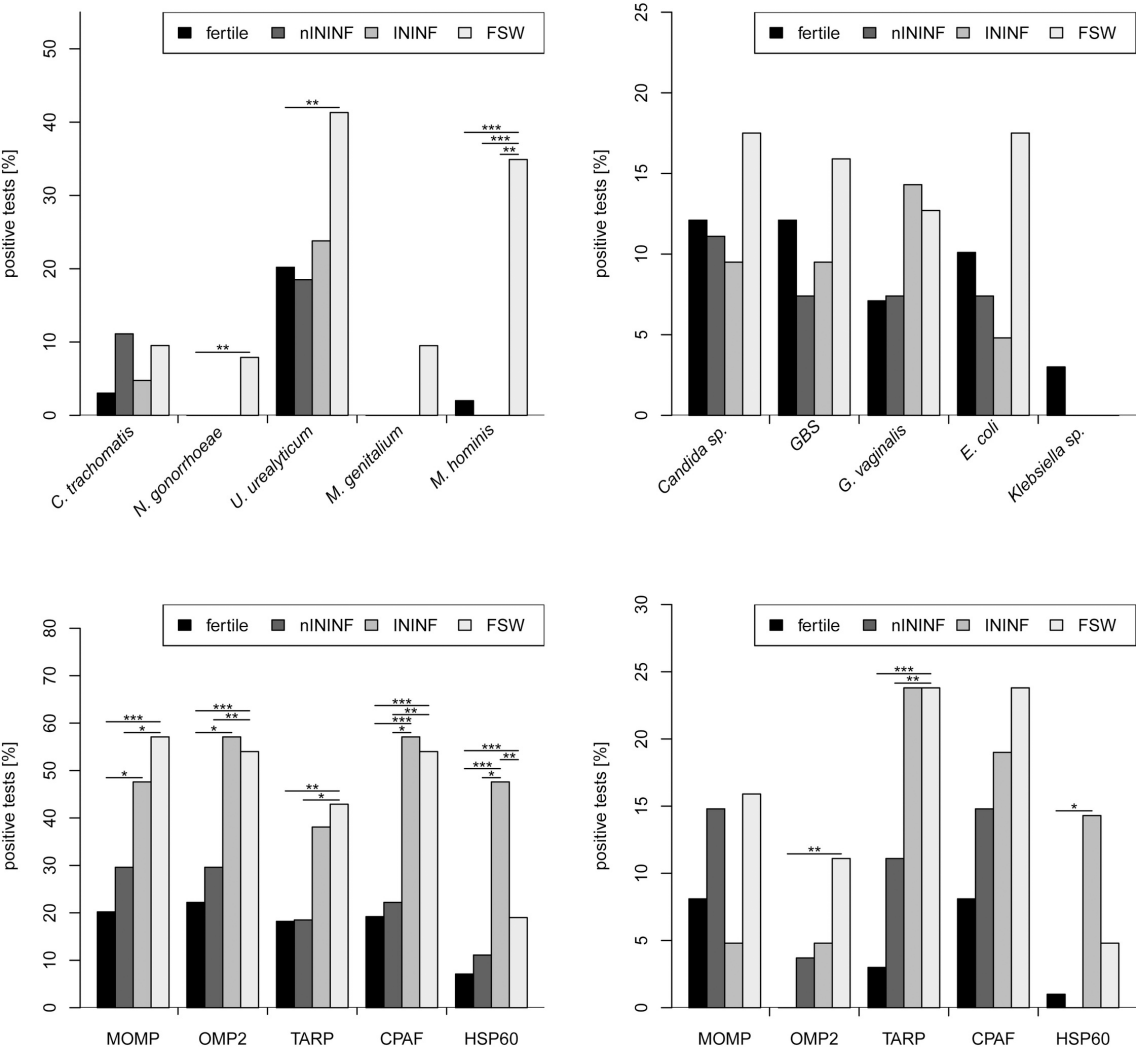
Results of the diagnostic testing for sexually transmitted infections. Diagnostics were performed by PCR (A) and conventional bacterial culture (B) from cervical swabs. Serological testing was apllied for IgG (C) and IgA (D) antibodies targeting different epitopes (MOMP, OMP2, TARP, CPAF and HSP60) of *C*. *trachomatis* (Fisher´s exact test: *p<0.05, **p<0.01, ***p<0.001). GBS: Group B *Streptococcus*.

Cervical swabs were subjected to standard culture of pathogenic bacteria and PCR-testing of *C*. *trachomatis*, *M*. *genitalium*, *M*. *hominis*, *U*. *urealyticum/parvum* and *N*. *gonorrhoeae*. Despite an increased rate of testing positive for *U*. *urealyticum/parvum* (41.30%), *N*. *gonorrhoeae* (7.90%), *M*. *genitalium* (9.50%) and *M*. *hominis* (34.90%) in FSW, no significant differences are observed between the other groups (Fig 2A and 2B).

### Serum antibodies against specific *C*. *trachomatis* epitopes differ in infectious infertility

We next tested *C*. *trachomatis* antibodies against IgG and IgA (S1 Fig) and performed immunoblotting against the chlamydial proteins MOMP, OMP2, TARP, CPAF and HSP60. In contrast to fertile controls, females with ININF have IgG antibodies targeting chlamydial antigens MOMP, OMP2, CPAF and HSP60 (Fig 2C) significantly more often. IgG antibodies against CPAF and HSP60 are also significantly enhanced in ININF when compared to nININF. FSW exhibit significantly higher frequencies in the detection of IgG antibodies against MOMP, OMP2, TARP and CPAF compared to both fertile controls and nININF. HSP60 IgG antibodies discriminate ININF from FSW showing significant reduction in FSW. In addition, significantly higher frequencies of IgA antibodies against OMP2 and TARP are observed for FSW, and OMP2 and HSP60 for ININF, when compared to the fertile controls (Fig 2D).

### Characterization of the cervical microbiota in infectious infertility

**Fig 3:**
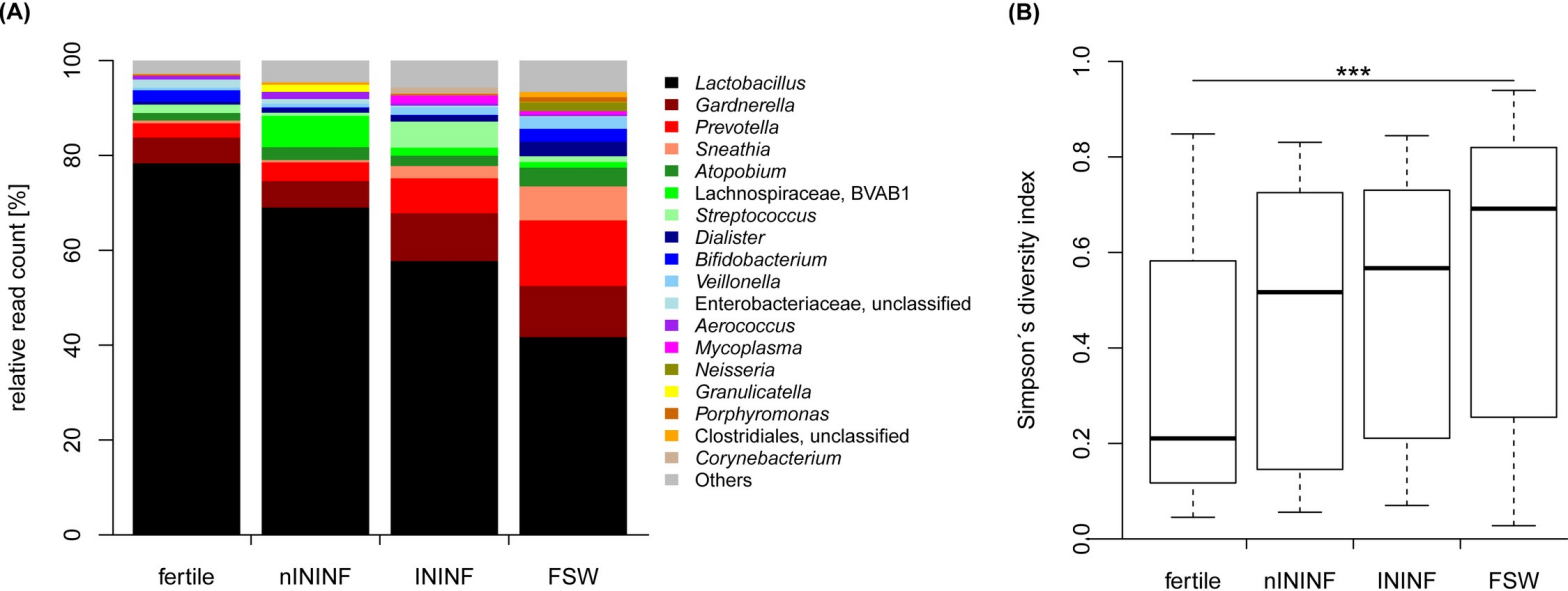
Average relative read count of bacteria and bacterial diversity differ between the study groups. The relative read count of genera in each study group is displayed (A). Genera are shown if they account for a minimum of one percent in at least one of the groups. An overview of statistical comparisons among taxa can be found in the S1 Table. The diversity of each sample was calculated using Simpson´s diversity index and plotted according to the study groups (B; Wilcoxon rank-sum test with Hochberg correction: ***p<0.001). nININF: Non-infectious infertility; ININF: Infectious infertility; FSW: Female sex worker.

**Fig 4:**
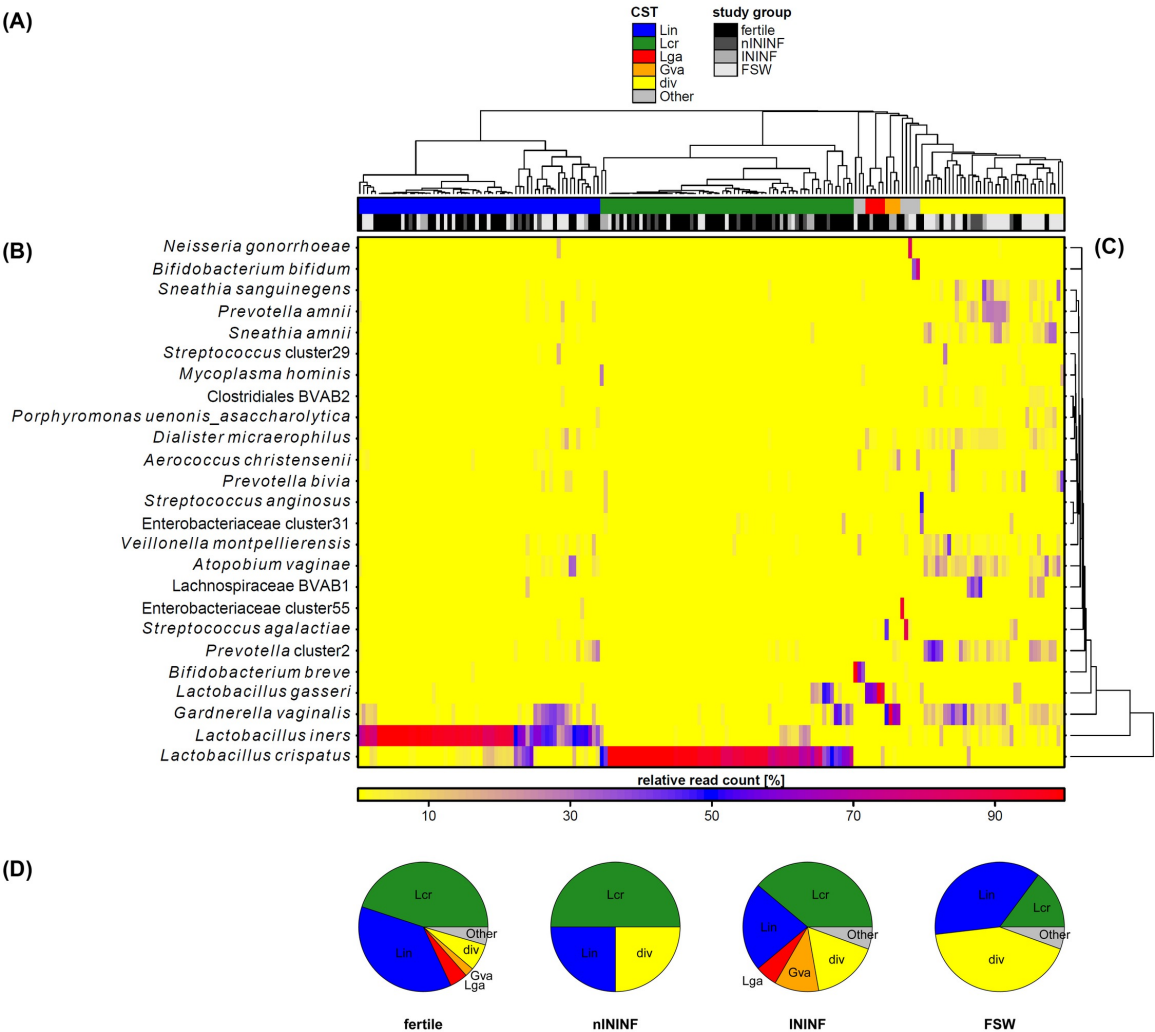
Women harbor several community state types (CST) that are differentially distributed between the study groups. Complete linkage clustering of samples based on the relative read count of bacteria and color key for study group assignment and CST (A). Heatmap showing relative read counts of the 25 most abundant bacterial taxa (B). Complete linkage clustering of the 25 most abundant bacterial taxa based on Spearman´s correlation coefficient (C). The samples cluster into CSTs and are named based on the respective dominant species as follows: Lcr: *L*. *crispatus*-dominated CST; Lin: *L*. *iners*-dominated CST; Lga: *L*. *gasseri*-dominated CST; Gva: *G*. *vaginalis*-dominated CST; div: diverse communities. Some single communities differ from these major community types and are comprised as Other. The distribution of the CSTs within study groups is shown in (D) (chi-square test: p<0.001). nININF: Non-infectious infertility; ININF: Infectious infertility; FSW: Female sex worker.

To further discriminate the local microbiota composition of the four different groups, we performed 16S rRNA gene sequencing of the V3/V4 hypervariable region from cervical swabs (Fig 3 and Fig 4). Comparing the average relative read count of genera in each study group, *Lactobacillus* is the most prominent genus. While on average 78.34% of all sequence reads in fertile females belong to *Lactobacillus*, the average percentage is reduced to 69.01% in nININF, 57.74% in ININF and 41.70% in FSW. In contrast, the relative read count of the genus *Gardnerella* increased from 5.43% in fertile females, to 5.61% in nININF and up to 10.08% in ININF and 10.78% in FSW. The same trend is observed for the genera *Prevotella* and *Sneathia* (Fig 3A). Some genera exhibit high detection rates in some single patients (e.g. *Mycoplasma* in ININF), while others are found frequently in moderate range in many patients (*Clostridiales* in FSW). However, this often allows the characterization down to the species level. Accordingly, all sequencing reads of *Neisseria* belong to *N*. *gonorrhoeae* and are exclusively found in FSW, whereas *Mycoplasma* reads could be attributed in more than 90% of the cases to *M*. *hominis*.

We specifically analyzed the sequencing results of those pathogens that were detected by conventional PCR-testing in a second swab. In contrast to the PCR-testing, the relative read count of *C*. *trachomatis* reads, as determined by microbiota sequencing, change. The mean relative read count of positively tested samples in fertile women, nININF and ININF is 0.14%, while the mean relative read count in FSW is 2.91% in positively tested samples.

An overview of statistical comparisons among taxa is given in the S1 Table. The diversity of each sample was calculated using Simpson´s diversity index (Fig 3B). The diversity is low in fertile females, with a median of 0.21, and higher in nININF (median: 0.52) and ININF (median: 0.57). FSW display the highest diversity indices with a median of 0.69. We observe an increase in diversity communities in groups with high sexual activity (S3 Fig), and a significant contribution of sexual activity to the alpha diversity (S2 Fig and S2 Table, R^2^ = 0.12, p<0.001).

**Fig 5:**
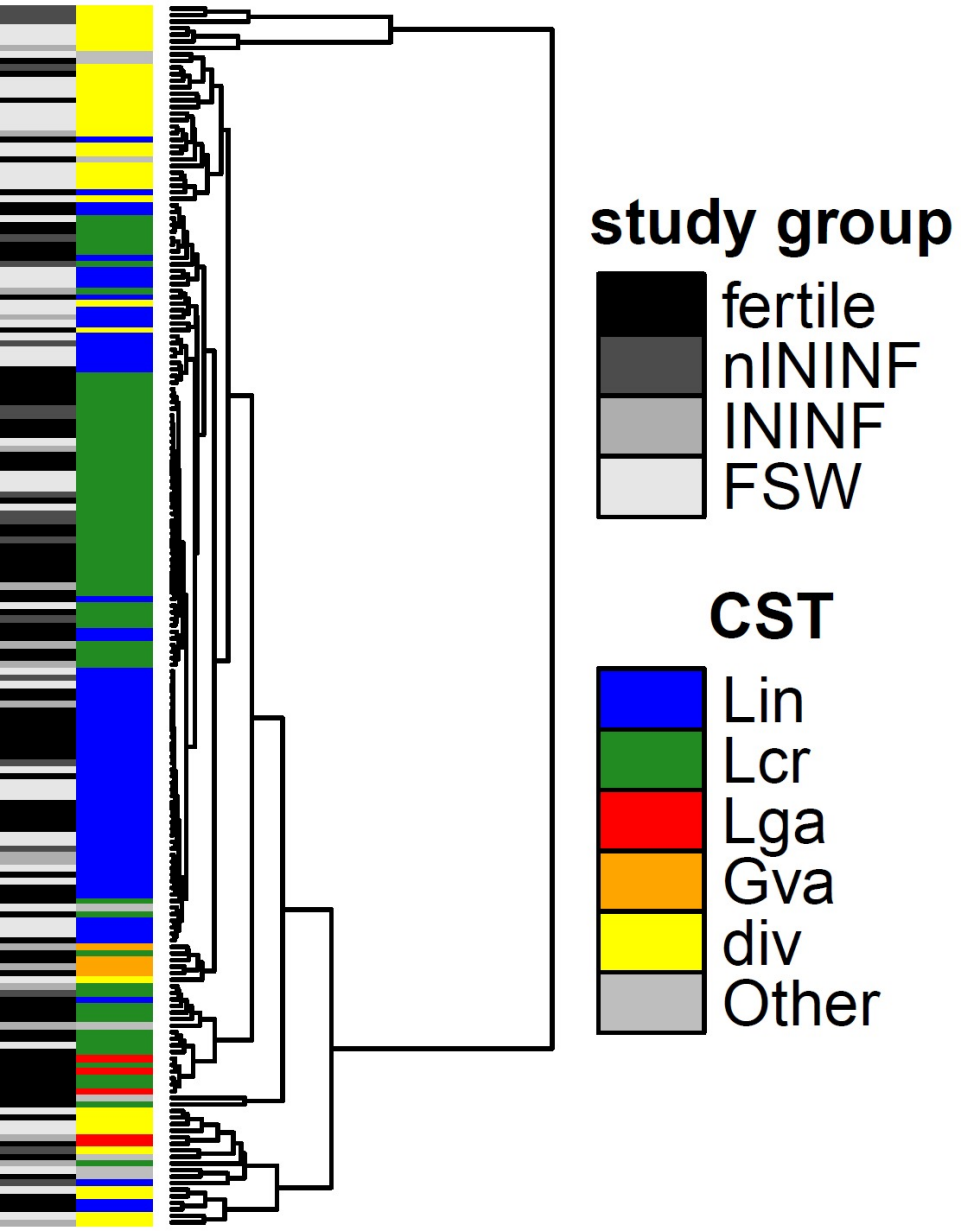
Metagenomic analysis using PICRUSt prediction of the metagenome based on normalized 16S rRNA gene sequences. Complete linkage clustering of samples based on predicted metagenomes and assignment of CSTs and study groups. The predicted metagenome clustering shows high concordance with the CST assignment. Lcr: *L*. *crispatus*-dominated CST; Lin: *L*. *iners*-dominated CST; Lga: *L*. *gasseri*-dominated CST; Gva: *G*. *vaginalis*-dominated CST; div: diverse communities. nININF: Non-infectious infertility; ININF: Infectious infertility; FSW: Female sex worker.

An established method for further characterization of differences in the microbial composition of the female urogenital tract is the classification in so called community state types (CST) [5]. Several CSTs can be attributed to the different study groups, showing greatly different relative read counts among taxa. The observed CSTs are dominated by *L*. *crispatus* (Lcr), *L*. *iners* (Lin), *L*. *gasseri* (Lga) or *G*. *vaginalis* (Gva), or displaying diverse community (div, Fig 4A–4D). Some single communities differ from these major CSTs (Other). Thus, fertile females mostly are comprised of *Lactobacillus*-dominated CSTs, with only low numbers of diverse communities or the Gva CST. The proportion of diverse communities is enhanced in nININF and INNIF, but in contrast to ININF, no Lga or Gva CST is observed in nININF. FSW are mostly colonized with diverse communities and the Lin CST, with only a limited number of the Lcr CST (Fig 4D, chi-square-test: p<0.001). While the Lcr CST is reduced in ININF and FSW compared to the other groups, the proportion of the Lin CST is relatively stable. Complete linkage clustering of samples based on predicted metagenomes and assignment of community state types display a high concordance (Fig 5).

### A statistical model for the prediction of infectious infertility

We further established a model to predict the underlying cause of infertility by using the following parameters: 1) detection of potential pathogens by PCR and cultivation, 2) serological status of *C*. *trachomatis* IgG/IgA and 3) the first ten taxa found in microbiota sequencing. Using binary logistic regression, we are able to define infectious infertility with an overall accuracy of 89.8% within the patient samples used in this study. We correctly predict 17 of 18 women from the ININF group, while all women from the nININF group are correctly separated from ININF. The ROC curve, assessing true *vs*. false positive rate with an area under curve of 0.978 proofs the performance of the model (S4 Fig). The same model, excluding microbiota results, predicts only 13 of 18 women to be correctly classified ININF, while the number of false positive predictions increases (for details and complete validation see S3 Table and S4 Fig).

## Discussion

Identification of females suffering from an infectious cause of infertility is arduous and has been based in the past on a combination of parameters including patient history, conventional microbial testing and gynecological assessments of anomalies of the reproductive tract. One of the major obstacles is the long time period between the acute initial exposure to a sexually transmitted pathogen and the individual recognition of a female becoming not pregnant later in life.

Although frequencies of STIs among adults are in the range of 1–10%, the public awareness in many countries is still low. Epidemiologic studies on *Chlamydia trachomatis* as the most frequent STI worldwide reveal high inter-study variations concerning its prevalence in European countries ranging from below 1% up 12% with confidence intervals ranging up to 20% [25, 26]. While PCR-based methods for the detection of acute infections are well established the usefulness of determining *C*. *trachomatis* antibody responses for diagnosing subsequent Chlamydia- derived sequelae are less certain. Detection of high antibody levels against the chlamydial-protease like activity factor (CPAF) in females with *Chlamydia trachomatis* cervicitis was first reported by Sharma et al., suggesting that CPAF is immunogenic during ascending chlamydial infection [27]. Forsbach-Birk et al. previously analyzed sera from 13 female patients with upper genital tract infections, and detection of *C*. *trachomatis* infection was confirmed by a positive PCR result and/or serum positivity of IgM antibodies. In this group MOMP, CPAF, OMP2, TARP and PmpD showed the highest overall diagnostic sensitivity and specificity [28]. In contrast to the findings in acute infections, our results highlight a role of HSP60, CPAF and OMP2 in host-pathogen interactions in females with ININF. In particular, HSP60 IgG antibodies separate the ININF group from all other groups, including FSW. Chlamydial HSP60 has been shown to be associated with PID [29, 30] which might explain why ININF display higher antibody titers against HSP60 than FSW in our study. Interestingly, antibodies against HSP60 and CPAF were also detected at significantly higher levels in inflammatory ocular *C*. *trachomatis* infections, suggesting both as markers for disease severity [31]. In addition, IgG antibodies against CPAF were significantly elevated among the trachomatous trichiasis cases, which is seen as a consequence of chronic inflammation in the tarsal conjunctiva [31].

Microbiota sequencing of the V3/V4 hypervariable region of the 16s rRNA gene from cervical swabs revealed further characteristic differences between the groups. Thus we detected significant differences in the relative read count of specific taxa, the diversity of the microbial communities and the proportions of the community types. In particular, genera (*Gardnerella*, *Prevotella*, and *Sneathia)* that have been linked to BV, together with *Atopobium*, *Peptostreptococcus*, *Mobiluncus*, *Leptotrichia*, *Mycoplasma*, and BV-associated bacterium 1 (BVAB1) to BVAB3 [3], are increased in the ININF and FSW group. This is accompanied by a shift in the vaginal community from the dominant *Lactobacillus* to a polymicrobial community [3]. Some of the above mentioned taxa, including *Dialister* and *Veillonella* were shown to be capable of producing biogenic amines, which might favor the development of a vaginal dysbiosis, but also could be used as a biomarker for BV detection [32]. Interestingly, while total *Lactobacillus-*dominated CSTs are reduced in ININF and FSW in comparison to fertile females and nININF, this is not true for the Lin CST, which is distributed rather equally among the study groups. This is of particular interest, as van der Veer et al. have recently shown that diverse bacterial communities (often containing high relative abundance of *G*. *vaginalis*) and vaginal communities dominated by *L*. *iners* are associated with *C*. *trachomatis* transmission [10]. Thus, ININF and FSW comprise high numbers of CSTs showing to prevent infections that cause infertility.

In interpreting microbiota composition of individuals, it must be considered that some genera might exhibit prominent detection rates in single groups because of high read numbers in some patients, while others are found frequently in a moderate range in many patients of the respective group. However, this often allows for the characterization down to the species level, and thereby greatly complements single-/multiplex-PCR approaches for respective STIs. Significantly higher loads of *M*. *hominis* were detected in women with BV, and the respective loads in *M*. *hominis* and *G*. *vaginalis* co-infections displayed a significant positive correlation [33].

Our data show that while *C*. *trachomatis* prevalence is equally distributed among the study groups, as according to the diagnostic PCR-testing, the relative read count in FSW is higher compared to the other groups in the microbiota data. This result further supports the hypothesis that direct detection of pathogenic bacteria in cervical swabs is not a good predictor for infections of the upper female genital tract. However, it would be interesting to see whether changes in relative read count upon acute infection might indicate a more severe disturbance of the physiological community and represent a risk factor for more severe disease. This hypothesis is supported by the finding that diverse bacterial communities are associated with *C*. *trachomatis* transmission [10].

The primers we used amplifying the V3/V4 regions were shown to be a good option with a high overall coverage for bacterial sequences (89.0%) with the lowest number of undetectable phyla of all primer pairs used in 16S microbiota assessment using 16S rRNA gene sequencing projects [34]. Notably, the typically undetected phyla for this primer pair are not members of the vaginal microbiota. This is a major advantage over the V1/V2 region, which was used in several key vaginal microbiota studies [6, 35], as this primer combination at best detects a limited amount of *Bifidobacteriales* (which includes the genus *Gardnerella*) and *Chlamydiales* [36], which are important in vaginal health and disease and play a prominent role in our urogenital community data.

We further analyzed whether the frequency of sexual intercourse has an impact on the microbial diversity, as an increase in sexual intercourse was proposed to induce a week negative effect on the stability of microbial communities [35]. It was reported in a group of African females that recent sexual exposure negatively affects the presence of *Lactobacillus* sp. and that having more than one sexual partner in the last three months significantly increases the prevalence of detecting *G*. *vaginalis* and *L*. *iners* [37]. In an observational study, Fethers at al. investigated the association between sexual risk factors and the occurrence of BV as determined by conventional techniques like the Amsel or a Gram stain method [38]. They observed that the epidemiological profile of BV was similar to that of established sexually transmitted infections. Bacterial vaginosis could also be linked with viral sexually transmitted infections (including HPV) in a univariate analysis. However, after controlling for sexual behaviour, the association was only significant for HSV1/HSV2 [39]. Although we observe a significant contribution of sexual activity to alpha diversity, the effect of increasing sexual intercourse is rather small and does not explain the variability in the data set.

Taken together, our data show that single parameters alone are not useful for distinguishing between infectious- and noninfectious infertility. Therefore we established a model for the prediction of ININF using binary logistic regression. The combination of several parameters, including microbiota sequencing data from cervical swabs significantly enhances the predictive power and discriminates all females suffering from noninfectious infertility. Further studies will be necessary on a larger scale to validate these findings and further refine our model in a clinical setting. Enhancing the number of ININF cases in the model building in future will increase its stability. Our results also highlight the potential of modern sequencing technologies to optimize classical microbiological diagnostic traits.

## Supporting information

S1 Fig Comparison of ELISA and immunoblot of IgG and IgA antibodies against *C*. *trachomatis*. (TIF)

S2 Fig Scatter plot showing the frequencies of sexual intercourse and the corresponding Shannon´s diversity indices. (TIF)

S3 Fig Distribution of community types dependent on the frequency of sexual intercourse per month. Increased sexual intercourse reduced the proportion of *L*. *crispatus* dominated community types while the number of diverse communities increased.
(TIF)

S4 Fig ROC curves describing true positive rate versus false positive rate of the binary logistic regression models predicting infectious infertility. Modelling including bacterial detection by PCR/culture and serology against *C*. *trachomatis* antigens (A) shows an area under curve of 0.847 (95% CI: 0.739–0.955) while the performance of the modelling improves when the microbiome data is integrated (B) with an area under curve of 0.978 (95% CI: 0.958–0.999).
(TIF)

S1 Table Comparison of the relative abundance of genera. (PPTX)

S2 Table Pearson´s product-moment correlation using Shannon´s diversity index as response and frequency of sexual intercourse/month as predictor. (PPTX)

S3 Table Prediction of the diagnosis “infectious infertility” using binary logististic regression. The number of positive predictions is given for different combinations of predictors in the groups fertile, nININF and ININF. Total accuracy and McFadden R^2^ are displayed.
(PPTX)

## References

[1] Fertility: Assessment and Treatment for People with Fertility Problems. National Institute for Health and Clinical Excellence: Guidance. London: National Institute for Health and Care Excellence 2013.

[2] Spiegel CA. Bacterial vaginosis. Reviews in Medical Microbiology. 2002;13(2):43–51.

[3] Onderdonk AB, Delaney ML, Fichorova RN. The Human Microbiome during Bacterial Vaginosis. Clin Microbiol Rev. 2016;29(2):223–38. doi: 29/2/223 [pii]; doi: 10.1128/CMR.00075-15 26864580 10.1128/CMR.00075-15 PMC4786887

[4] Muzny CA, Sunesara IR, Kumar R, Mena LA, Griswold ME, Martin DH, et al Characterization of the vaginal microbiota among sexual risk behavior groups of women with bacterial vaginosis. PLoS One. 2013;8(11):e80254 doi: 10.1371/journal.pone.0080254 ; PubMed Central PMCID: PMC3827412. 24236175 10.1371/journal.pone.0080254 PMC3827412

[5] DiGiulio DB, Callahan BJ, McMurdie PJ, Costello EK, Lyell DJ, Robaczewska A, et al Temporal and spatial variation of the human microbiota during pregnancy. Proc Natl Acad Sci U S A. 2015;112(35):11060–5. doi: 10.1073/pnas.1502875112 26283357 10.1073/pnas.1502875112 PMC4568272

[6] Ravel J, Gajer P, Abdo Z, Schneider GM, Koenig SSK, McCulle SL, et al Vaginal microbiome of reproductive-age women. Proc Natl Acad Sci U S A. 2011;108:4680–7. doi: 10.1073/pnas.1002611107 20534435 10.1073/pnas.1002611107 PMC3063603

[7] Ghartey JP, Smith BC, Chen Z, Buckley N, Lo Y, Ratner AJ, et al Lactobacillus crispatus dominant vaginal microbiome is associated with inhibitory activity of female genital tract secretions against Escherichia coli. PLoS One. 2014;9(5):e96659 doi: 10.1371/journal.pone.0096659 PONE-D-13-36660 [pii]. 24805362 10.1371/journal.pone.0096659 PMC4013016

[8] Nunn KL, Wang YY, Harit D, Humphrys MS, Ma B, Cone R, et al Enhanced Trapping of HIV-1 by Human Cervicovaginal Mucus Is Associated with Lactobacillus crispatus-Dominant Microbiota. MBio. 2015;6(5):e01084–15. doi: mBio.01084-15 [pii]; doi: 10.1128/mBio.01084-15 26443453 10.1128/mBio.01084-15 PMC4611035

[9] Nardini P, Nahui Palomino RA, Parolin C, Laghi L, Foschi C, Cevenini R, et al Lactobacillus crispatus inhibits the infectivity of Chlamydia trachomatis elementary bodies, in vitro study. Sci Rep. 2016;6:29024. doi: srep29024 [pii]; doi: 10.1038/srep29024 27354249 10.1038/srep29024 PMC4926251

[10] van der Veer C, Bruisten SM, van der Helm JJ, de Vries H, van Houdt R. The cervico-vaginal microbiota in women notified for Chlamydia trachomatis infection: A case-control study at the STI outpatient clinic in Amsterdam, the Netherlands. Clin Infect Dis. 2016. doi: ciw586 [pii]; doi: 10.1093/cid/ciw586 27567124 10.1093/cid/ciw586

[11] Ziklo N, Huston WM, Hocking JS, Timms P. Chlamydia trachomatis Genital Tract Infections: When Host Immune Response and the Microbiome Collide. Trends Microbiol. 2016;24(9):750–65. doi: S0966-842X(16)30048-8 [pii]; doi: 10.1016/j.tim.2016.05.007 27320172 10.1016/j.tim.2016.05.007

[12] Kozich JJ, Westcott SL, Baxter NT, Highlander SK, Schloss PD. Development of a dual-index sequencing strategy and curation pipeline for analyzing amplicon sequence data on the MiSeq Illumina sequencing platform. Appl Environ Microbiol. 2013;79(17):5112–20. doi: AEM.01043-13 [pii]; doi: 10.1128/AEM.01043-13 23793624 10.1128/AEM.01043-13 PMC3753973

[13] Schloss PD, Westcott SL, Ryabin T, Hall JR, Hartmann M, Hollister EB, et al Introducing mothur: open-source, platform-independent, community-supported software for describing and comparing microbial communities. Appl Environ Microbiol. 2009;75(23):7537–41. doi: AEM.01541-09 [pii]; doi: 10.1128/AEM.01541-09 19801464 10.1128/AEM.01541-09 PMC2786419

[14] Pruesse E, Quast C, Knittel K, Fuchs BM, Ludwig WG, Peplies J, et al SILVA: a comprehensive online resource for quality checked and aligned ribosomal RNA sequence data compatible with ARB. Nucleic Acids Research. 2007;35(21):7188–96. doi: 10.1093/nar/gkm864 17947321 10.1093/nar/gkm864 PMC2175337

[15] Edgar RC, Haas BJ, Clemente JC, Quince C, Knight R. UCHIME improves sensitivity and speed of chimera detection. Bioinformatics. 2011;27(16):2194–200. doi: 10.1093/bioinformatics/btr381 21700674 10.1093/bioinformatics/btr381 PMC3150044

[16] Wang Q, Garrity GM, Tiedje JM, Cole JR. Naive Bayesian classifier for rapid assignment of rRNA sequences into the new bacterial taxonomy. Appl Environ Microbiol. 2007;73(16):5261–7. doi: 10.1128/AEM.00062-07 17586664 10.1128/AEM.00062-07 PMC1950982

[17] Fettweis JM, Serrano MG, Sheth NU, Mayer CM, Glascock AL, Brooks JP, et al Species-level classification of the vaginal microbiome. BMC Genomics. 2012;13. 10.1186/1471-2164-13-S8-S17 PMC3535711 23282177

[18] Langille MG, Zaneveld J, Caporaso JG, McDonald D, Knights D, Reyes JA, et al Predictive functional profiling of microbial communities using 16S rRNA marker gene sequences. Nature biotechnology. 2013;31(9):814–21. doi: 10.1038/nbt.2676 ; PubMed Central PMCID: PMC3819121. 23975157 10.1038/nbt.2676 PMC3819121

[19] Team RC. R: A language and environment for statistical computing R Foundation for Statistical Computing, Vienna, AustriaURL https://wwwR-projectorg/. 2015.

[20] Oksanen J, Blanchet FG, Kindt R, Legendre P, Minchin PR, O`Hara RB, et al. vegan: Community Ecology Package. R package version 23–2http://CRANR-projectorg/package=vegan. 2015.

[21] Sauerbrei W, Meier-Hirmer C, Benner A, Royston P. Multivariable regression model building by using fractional polynomials: Description of SAS, STATA and R programs. Computational Statistics & Data Analysis. 2006;50(12):3464–85.

[22] Boutros PC. BoutrosLab.plotting.general: Functions to Create Publication-Quality plots. R package version 534. 2015.

[23] Sing T, Sander O, Beerenwinkel N, Lengauer T. ROCR: visualizing classifier performance in R. Bioinformatics. 2005;21(20):3940–1. doi: 10.1093/bioinformatics/bti623 16096348 10.1093/bioinformatics/bti623

[24] Robin X, Turck N, Hainard A, Tiberti N, Lisacek F, Sanchez JC, et al pROC: an open-source package for R and S plus to analyze and compare ROC curves. Bmc Bioinformatics. 2011;12(77). 10.1186/1471-2105-12-77 PMC3068975 21414208

[25] Control ECfDPa. Chlamydia control in Europe: literature review. ECDC Technical Report. 2008.

[26] Dielissen PW, Teunissen DAM, Lagro-Janssen ALM. Chlamydia prevalence in the general population: is there a sex difference? a systematic review. BMC Infect Dis. 2013;13(534). 10.1186/1471-2334-13-534 PMC4225722 24215287

[27] Sharma J, Bosnic AM, Piper JM, Zhong G. Human antibody responses to a Chlamydia-secreted protease factor. Infect Immun. 2004;72(12):7164–71. doi: 72/12/7164 [pii]; doi: 10.1128/IAI.72.12.7164-7171.2004 15557641 10.1128/IAI.72.12.7164-7171.2004 PMC529132

[28] Forsbach-Birk V, Simnacher U, Pfrepper KI, Soutschek E, Kiselev AO, Lampe MF, et al Identification and evaluation of a combination of chlamydial antigens to support the diagnosis of severe and invasive Chlamydia trachomatis infections. Clin Microbiol Infect. 2010;16(8):1237–44. doi: 10.1111/j.1469-0691.2009.03041.x 19723133 10.1111/j.1469-0691.2009.03041.x

[29] Witkin SS, Minis E, Athanasiou A, Leizer J, Linhares IM. Chlamydia trachomatis: the Persistent Pathogen. Clinical and vaccine immunology: CVI. 2017;24(10). doi: 10.1128/CVI.00203-17 ; PubMed Central PMCID: PMC5629669. 28835360 10.1128/CVI.00203-17 PMC5629669

[30] Peeling RW, Kimani J, Plummer F, Maclean I, Cheang M, Bwayo J, et al Antibody to chlamydial hsp60 predicts an increased risk for chlamydial pelvic inflammatory disease. J Infect Dis. 1997;175(5):1153–8. . 9129079 10.1086/516454

[31] Skwor T, Kandel RP, Basravi S, Khan A, Sharma B, Dean D. Characterization of Humoral Immune Responses to Chlamydial HSP60, CPAF, and CT795 in Inflammatory and Severe Trachoma. Invest Ophthalmol Vis Sci. 2010;51(10):5128–36. doi: 10.1167/iovs.09-5113 20463311 10.1167/iovs.09-5113 PMC3066612

[32] Nelson TM, Borgogna JLC, Brotman RM, Ravel J, Walk ST, Yeoman CJ. Vaginal biogenic amines: biomarkers of bacterial vaginosis or precursors to vaginal dysbiosis? Frontiers in Physiology. 2015;6:253 doi: 10.3389/fphys.2015.00253 26483694 10.3389/fphys.2015.00253 PMC4586437

[33] Cox C, Watt AP, McKenna JP, Coyle PV. Mycoplasma hominis and Gardnerella vaginalis display a significant synergistic relationship in bacterial vaginosis. Eur J Clin Microbiol Infect Dis. 2016;35(3):481–7. doi: 10.1007/s10096-015-2564-x 26796553 10.1007/s10096-015-2564-x

[34] Klindworth A, Pruesse E, Schweer T, Peplies J, Quast C, Horn M, et al Evaluation of general 16S ribosomal RNA gene PCR primers for classical and next-generation sequencing-based diversity studies. Nucleic Acids Res. 2013;41(1):e1. doi: gks808 [pii]; doi: 10.1093/nar/gks808 22933715 10.1093/nar/gks808 PMC3592464

[35] Gajer P, Brotman RM, Bai GY, Sakamoto J, Schuette UME, Zhong X, et al Temporal Dynamics of the Human Vaginal Microbiota. Sci Transl Med. 2012;4(132):132–52. 10.1126/scitranslmed.3003605 PMC3722878 22553250

[36] Frank JA, Reich CI, Sharma S, Weisbaum JS, Wilson BA, Olsen GJ. Critical evaluation of two primers commonly used for amplification of bacterial 16S rRNA genes. Appl Environ Microbiol. 2008;74(8):2461–70. doi: AEM.02272-07 [pii]; doi: 10.1128/AEM.02272-07 18296538 10.1128/AEM.02272-07 PMC2293150

[37] Jespers V, van de Wijgert J, Cools P, Verhelst R, Verstraelen H, Delany-Moretlwe S, et al The significance of Lactobacillus crispatus and L. vaginalis for vaginal health and the negative effect of recent sex: a cross-sectional descriptive study across groups of African women. BMC Infect Dis. 2015;15(115). doi: 10.1186/s12879-015-0825-z 25879811 10.1186/s12879-015-0825-z PMC4351943

[38] Fethers KA, Fairley CK, Hocking JS, Gurrin LC, Bradshaw CS. Sexual Risk Factors and Bacterial Vaginosis: A Systematic Review and Meta-Analysis. Clin Infect Dis. 2008;47(11):1426–35. doi: 10.1086/592974 18947329 10.1086/592974

[39] Allsworth JE, Lewis VA, Peipert JF. Viral sexually transmitted infections and bacterial vaginosis: 2001-2004 national health and nutrition examination survey data. Sex Transm Dis. 2008;35(9):791–6. doi: 10.1097/OLQ.0b013e3181788301 . 18607314 10.1097/OLQ.0b013e3181788301

