# Supplementary figures and images for: Microbiota-based analysis reveals specific bacterial traits and a novel strategy for the diagnosis of infectious infertility
Source: PLoS One. 2018 Jan 9;13(1):e0191047. doi: 10.1371/journal.pone.0191047 (PMC5760088; doi:10.1371/journal.pone.0191047)

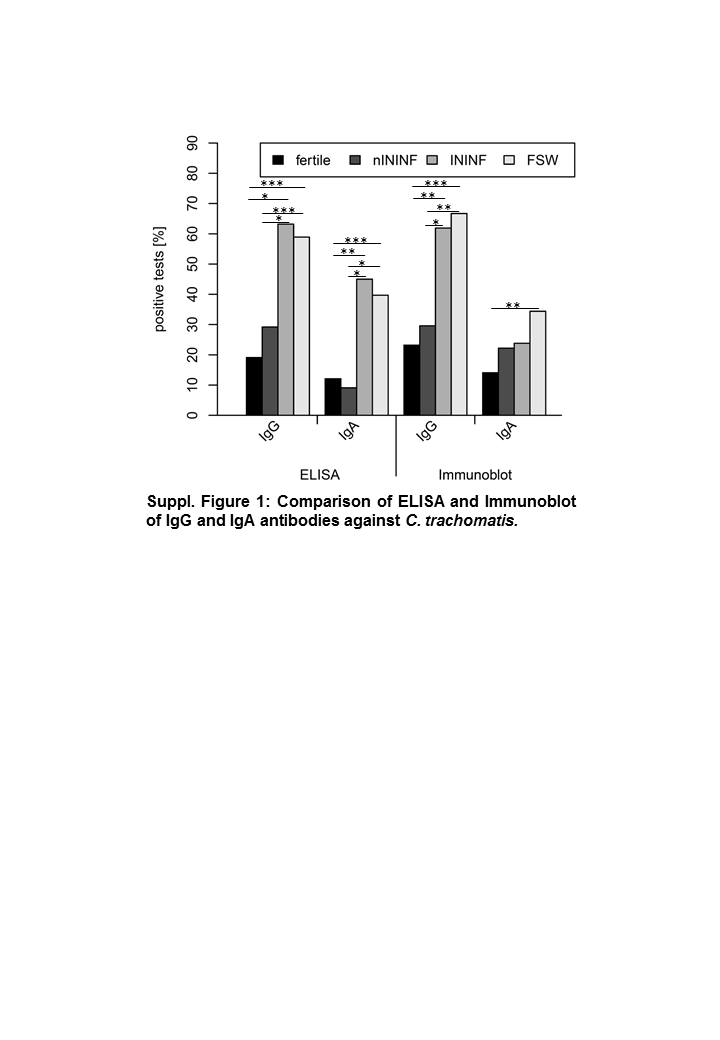

Supplement: S1 Fig — (TIF) [file pone.0191047.s001.TIF]

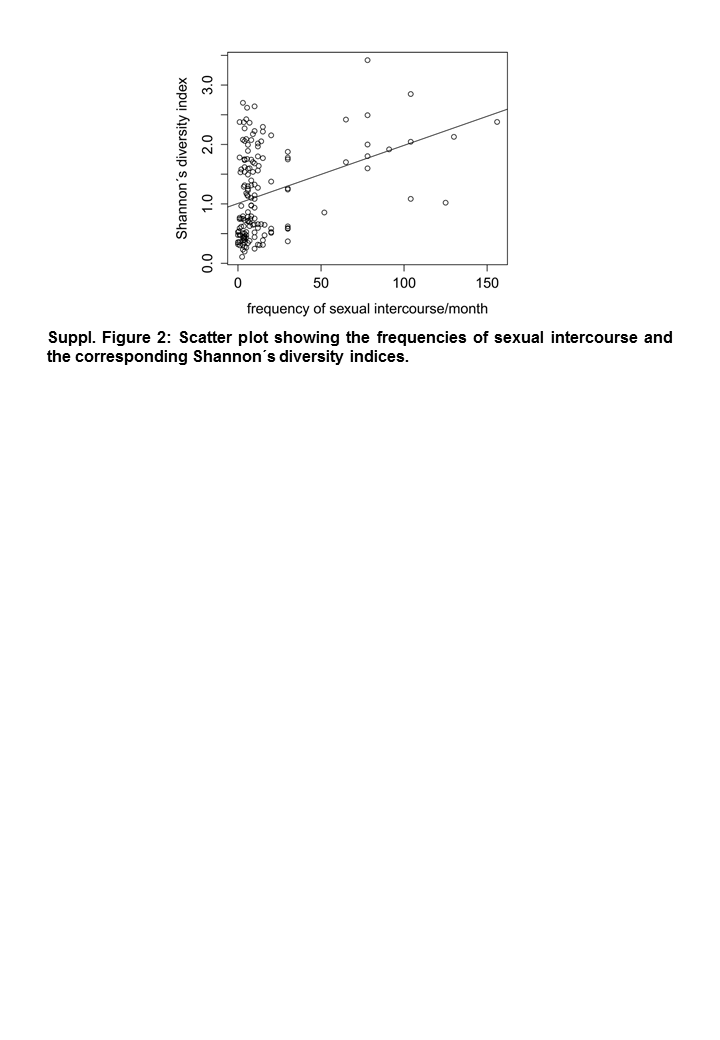

Supplement: S2 Fig — (TIF) [file pone.0191047.s002.TIF]

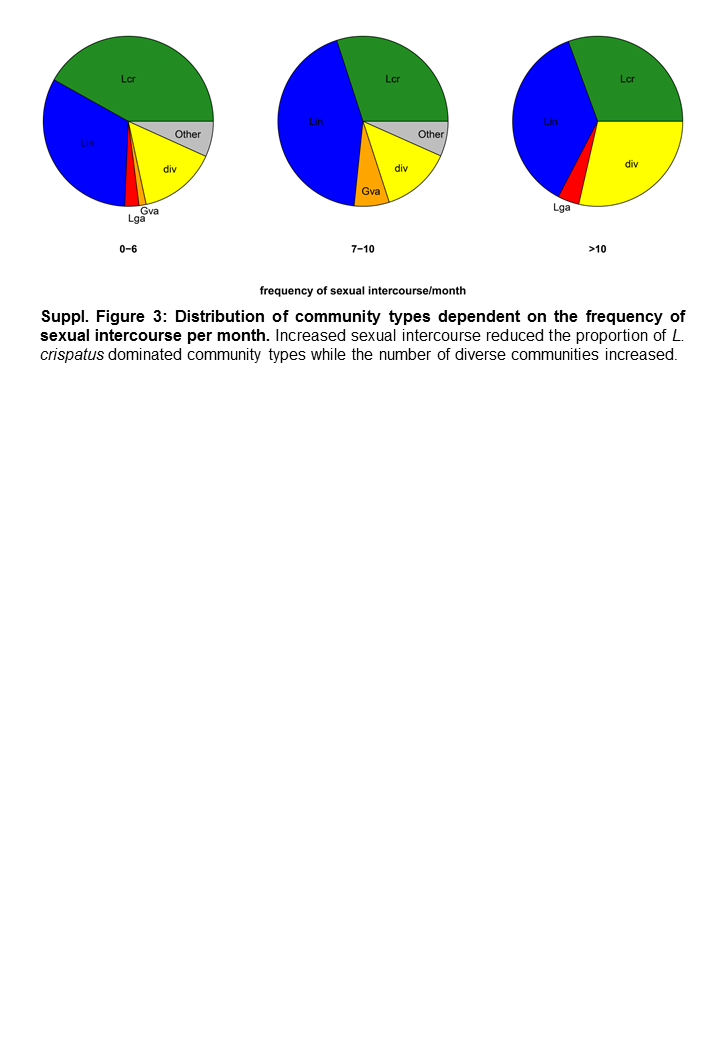

Supplement: S3 Fig — Increased sexual intercourse reduced the proportion of L. crispatus dominated community types while the number of diverse communities increased. (TIF) [file pone.0191047.s003.TIF]

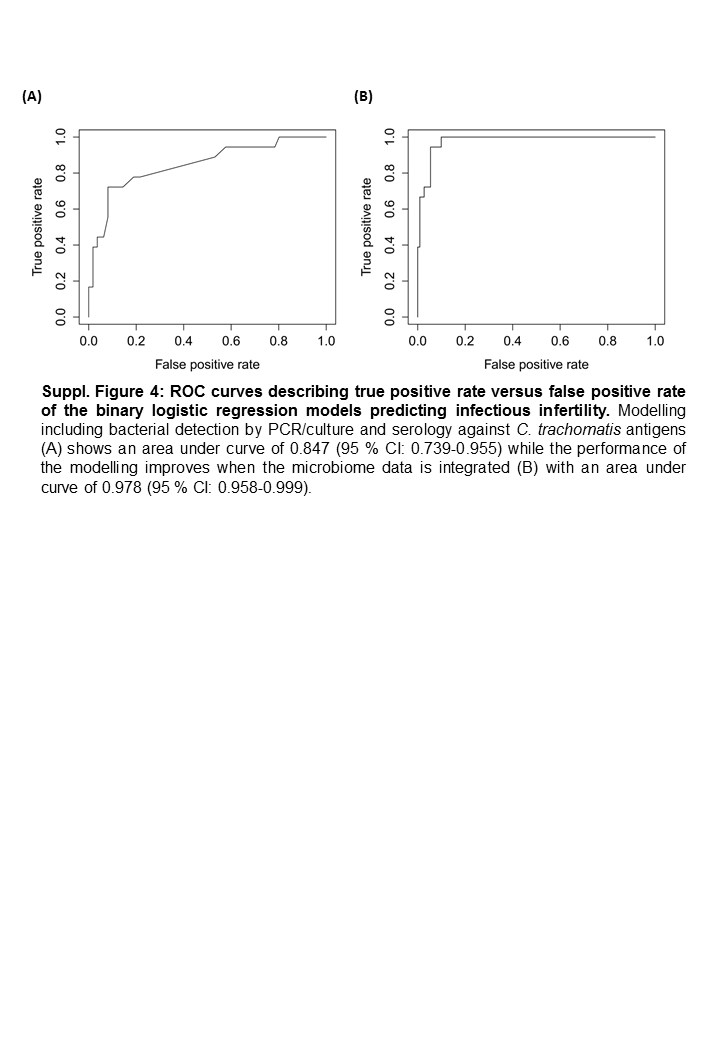

Supplement: S4 Fig — Modelling including bacterial detection by PCR/culture and serology against C. trachomatis antigens (A) shows an area under curve of 0.847 (95% CI: 0.739–0.955) while the performance of the modelling improves when the microbiome data is integrated (B) with an area under curve of 0.978 (95% CI: 0.958–0.999). (TIF) [file pone.0191047.s004.TIF]
