# Supplementary material for: Microbiota-based analysis reveals specific bacterial traits and a novel strategy for the diagnosis of infectious infertility
Source: PLoS One. 2018 Jan 9;13(1):e0191047. doi: 10.1371/journal.pone.0191047 (PMC5760088; doi:10.1371/journal.pone.0191047)
Supplement: S1 Table — (PPTX) [file pone.0191047.s005.pptx]

## Slide 1
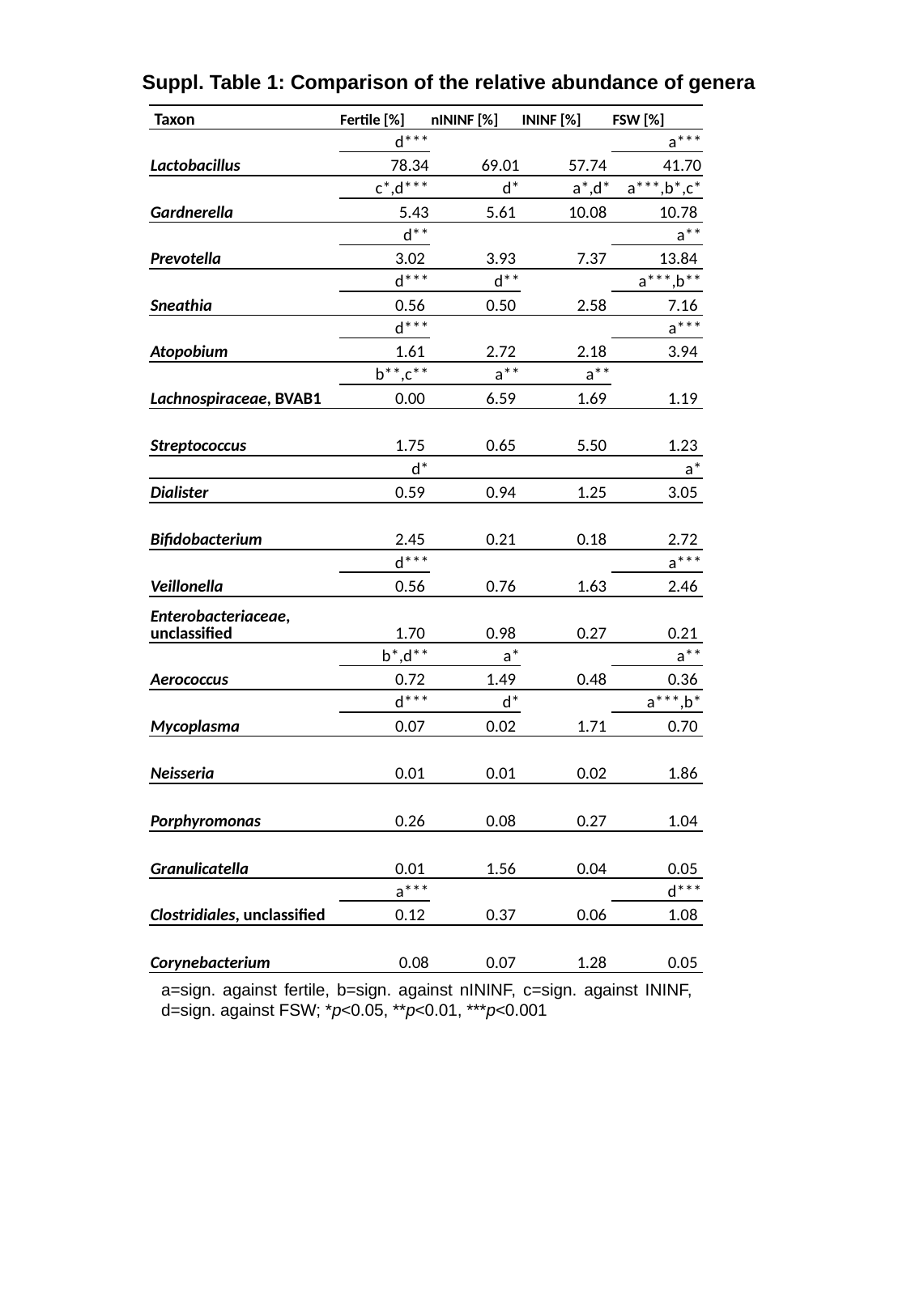

Suppl. Table 1: Comparison of the relative abundance of genera
| Taxon | Fertile [%] | nININF [%] | ININF [%] | FSW [%] |
| --- | --- | --- | --- | --- |
| Lactobacillus | d\*\*\* | 69.01 | 57.74 | a\*\*\* |
| | 78.34 | | | 41.70 |
| Gardnerella | c\*,d\*\*\* | d\* | a\*,d\* | a\*\*\*,b\*,c\* |
| | 5.43 | 5.61 | 10.08 | 10.78 |
| Prevotella | d\*\* | 3.93 | 7.37 | a\*\* |
| | 3.02 | | | 13.84 |
| Sneathia | d\*\*\* | d\*\* | 2.58 | a\*\*\*,b\*\* |
| | 0.56 | 0.50 | | 7.16 |
| Atopobium | d\*\*\* | 2.72 | 2.18 | a\*\*\* |
| | 1.61 | | | 3.94 |
| Lachnospiraceae, BVAB1 | b\*\*,c\*\* | a\*\* | a\*\* | 1.19 |
| | 0.00 | 6.59 | 1.69 | |
| Streptococcus | 1.75 | 0.65 | 5.50 | 1.23 |
| | d\* | | | a\* |
| Dialister | 0.59 | 0.94 | 1.25 | 3.05 |
| Bifidobacterium | 2.45 | 0.21 | 0.18 | 2.72 |
| Veillonella | d\*\*\* | 0.76 | 1.63 | a\*\*\* |
| | 0.56 | | | 2.46 |
| Enterobacteriaceae, unclassified | 1.70 | 0.98 | 0.27 | 0.21 |
| Aerococcus | b\*,d\*\* | a\* | 0.48 | a\*\* |
| | 0.72 | 1.49 | | 0.36 |
| Mycoplasma | d\*\*\* | d\* | 1.71 | a\*\*\*,b\* |
| | 0.07 | 0.02 | | 0.70 |
| Neisseria | 0.01 | 0.01 | 0.02 | 1.86 |
| Porphyromonas | 0.26 | 0.08 | 0.27 | 1.04 |
| Granulicatella | 0.01 | 1.56 | 0.04 | 0.05 |
| Clostridiales, unclassified | a\*\*\* | 0.37 | 0.06 | d\*\*\* |
| | 0.12 | | | 1.08 |
| Corynebacterium | 0.08 | 0.07 | 1.28 | 0.05 |
a=sign. against fertile, b=sign. against nININF, c=sign. against ININF, d=sign. against FSW; *p<0.05, **p<0.01, ***p<0.001
