# Supplementary material for: Microbiota-based analysis reveals specific bacterial traits and a novel strategy for the diagnosis of infectious infertility
Source: PLoS One. 2018 Jan 9;13(1):e0191047. doi: 10.1371/journal.pone.0191047 (PMC5760088; doi:10.1371/journal.pone.0191047)
Supplement: S2 Table — (PPTX) [file pone.0191047.s006.pptx]

## Slide 1
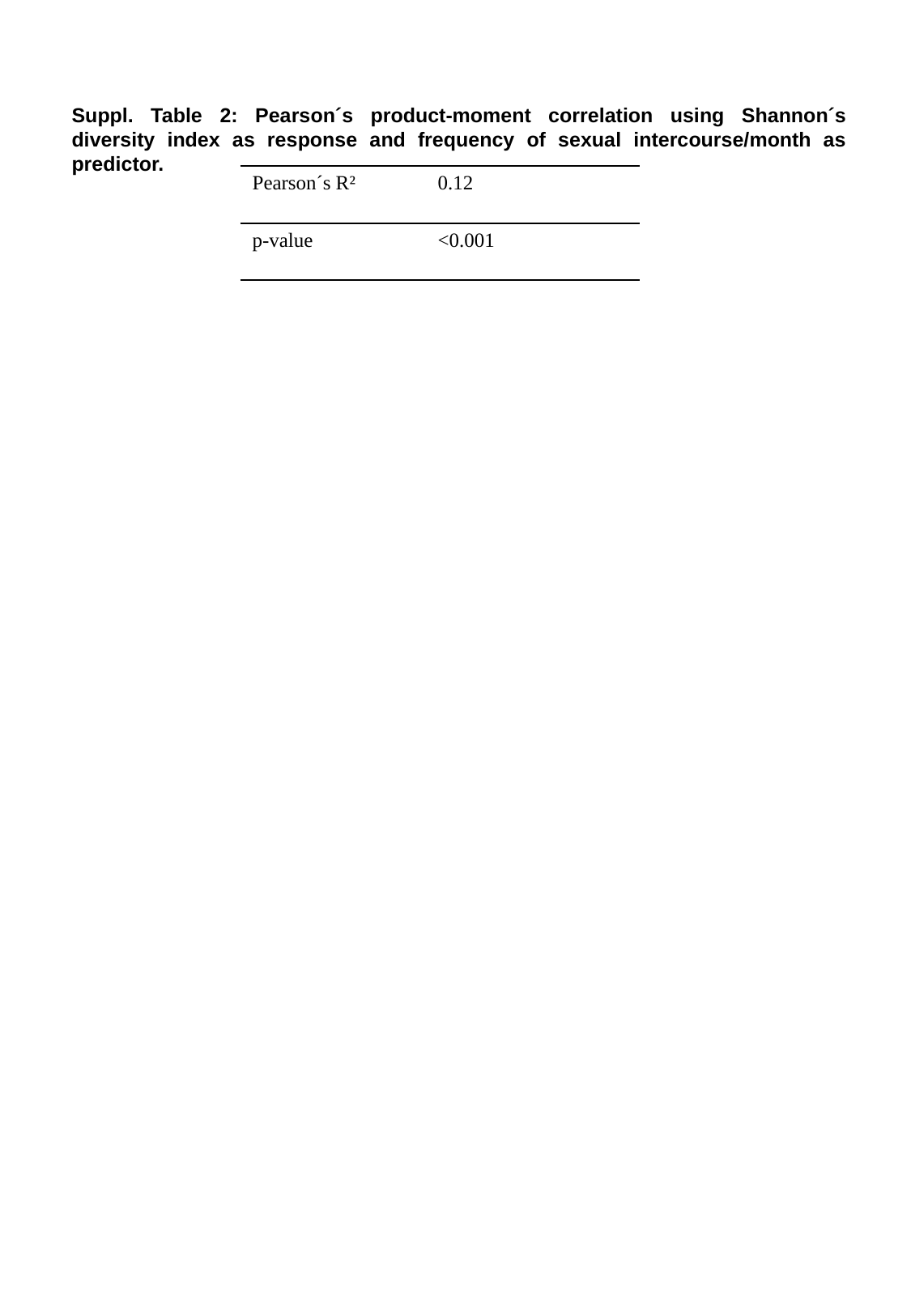

Suppl. Table 2: Pearson´s product-moment correlation using Shannon´s diversity index as response and frequency of sexual intercourse/month as predictor.
| Pearson´s R² | 0.12 |
| --- | --- |
| p-value | <0.001 |
