# Supplementary material for: Microbiota-based analysis reveals specific bacterial traits and a novel strategy for the diagnosis of infectious infertility
Source: PLoS One. 2018 Jan 9;13(1):e0191047. doi: 10.1371/journal.pone.0191047 (PMC5760088; doi:10.1371/journal.pone.0191047)
Supplement: S3 Table — The number of positive predictions is given for different combinations of predictors in the groups fertile, nININF and ININF. Total accuracy and McFadden R2 are displayed. (PPTX) [file pone.0191047.s007.pptx]

## Slide 1
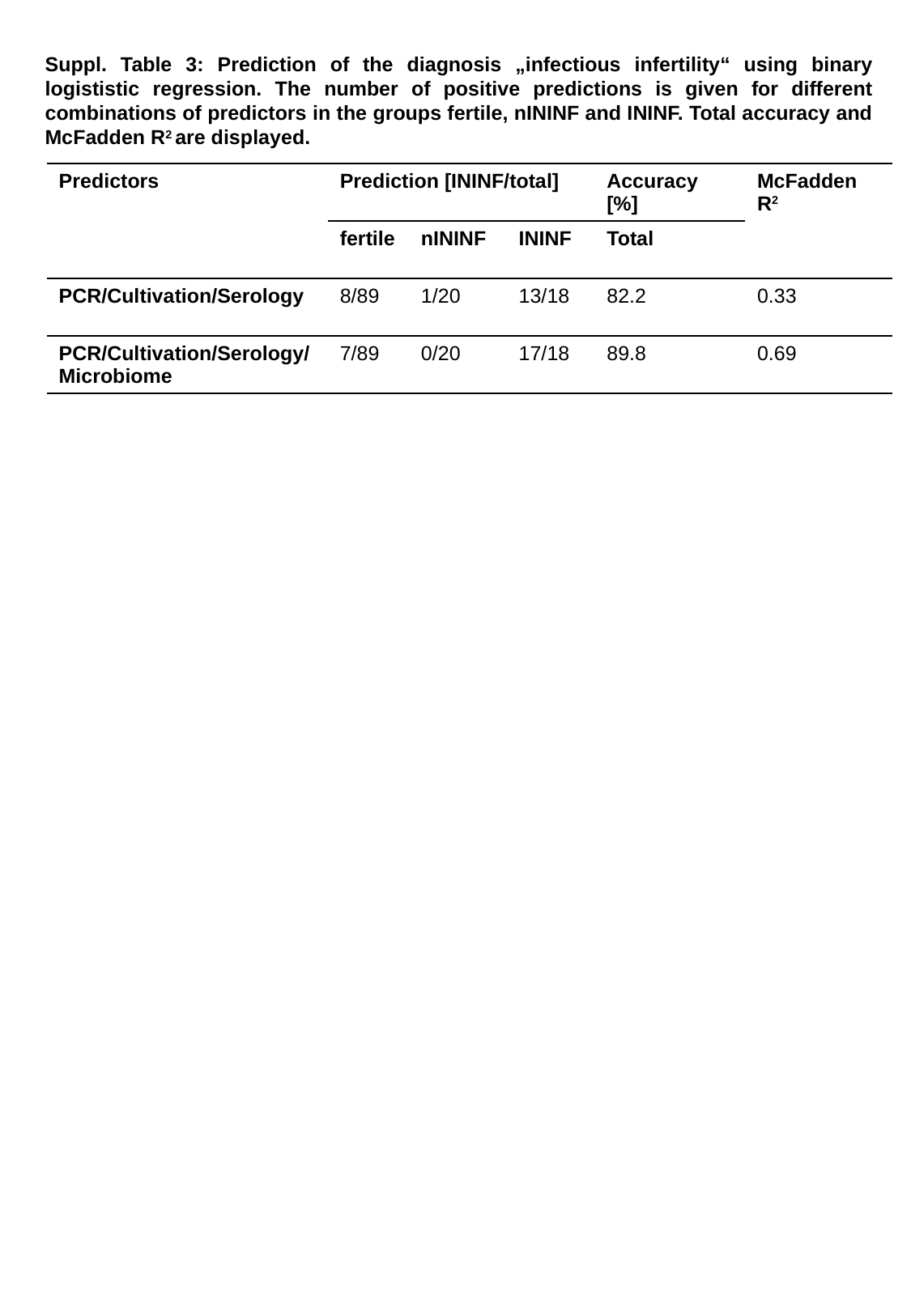

Suppl. Table 3: Prediction of the diagnosis „infectious infertility“ using binary logististic regression. The number of positive predictions is given for different combinations of predictors in the groups fertile, nININF and ININF. Total accuracy and McFadden R2 are displayed.
| Predictors | Prediction [ININF/total] | | | Accuracy [%] | McFadden R2 |
| --- | --- | --- | --- | --- | --- |
| | fertile | nININF | ININF | Total | |
| PCR/Cultivation/Serology | 8/89 | 1/20 | 13/18 | 82.2 | 0.33 |
| PCR/Cultivation/Serology/ Microbiome | 7/89 | 0/20 | 17/18 | 89.8 | 0.69 |
